# Biomarker-based classification of bacterial and fungal whole-blood infections in a genome-wide expression study

**DOI:** 10.3389/fmicb.2015.00171

**Published:** 2015-03-11

**Authors:** Andreas Dix, Kerstin Hünniger, Michael Weber, Reinhard Guthke, Oliver Kurzai, Jörg Linde

**Affiliations:** ^1^Systems Biology/Bioinformatics, Leibniz Institute for Natural Product Research and Infection Biology – Hans-Knöll-InstituteJena, Germany; ^2^Septomics Research Centre, Friedrich Schiller University and Leibniz Institute for Natural Product Research and Infection Biology – Hans-Knöll-InstituteJena, Germany

**Keywords:** immune response, microarray, feature selection, systems biology, decision tree based methods, fungal pathogens

## Abstract

Sepsis is a clinical syndrome that can be caused by bacteria or fungi. Early knowledge on the nature of the causative agent is a prerequisite for targeted anti-microbial therapy. Besides currently used detection methods like blood culture and PCR-based assays, the analysis of the transcriptional response of the host to infecting organisms holds great promise. In this study, we aim to examine the transcriptional footprint of infections caused by the bacterial pathogens *Staphylococcus aureus* and *Escherichia coli* and the fungal pathogens *Candida albicans* and *Aspergillus fumigatus* in a human whole-blood model. Moreover, we use the expression information to build a random forest classifier to classify if a sample contains a bacterial, fungal, or mock-infection. After normalizing the transcription intensities using stably expressed reference genes, we filtered the gene set for biomarkers of bacterial or fungal blood infections. This selection is based on differential expression and an additional gene relevance measure. In this way, we identified 38 biomarker genes, including *IL6, SOCS3*, and *IRG1* which were already associated to sepsis by other studies. Using these genes, we trained the classifier and assessed its performance. It yielded a 96% accuracy (sensitivities >93%, specificities >97%) for a 10-fold stratified cross-validation and a 92% accuracy (sensitivities and specificities >83%) for an additional test dataset comprising *Cryptococcus neoformans* infections. Furthermore, the classifier is robust to Gaussian noise, indicating correct class predictions on datasets of new species. In conclusion, this genome-wide approach demonstrates an effective feature selection process in combination with the construction of a well-performing classification model. Further analyses of genes with pathogen-dependent expression patterns can provide insights into the systemic host responses, which may lead to new anti-microbial therapeutic advances.

## 1. Introduction

Sepsis is a critical medical condition with high mortality rates. It is characterized by a dysregulation of the inflammatory response of the host due to a microbial infection. The uncontrolled inflammation can lead to tissue and organ damage, eventually resulting in death of the patient (Rittirsch et al., 2008). The incidence of sepsis has been increasing worldwide (Engel et al., 2007; Martin, 2012). In fact, sepsis is the 10th most common cause of death with a mortality rate of 20–50% in the US (Martin et al., 2003). The most frequent causative pathogens are bacteria, most commonly staphylococci and Enterobacteriaceae like *E. coli* (Martin, 2012). While the overall incidence of sepsis is increasing about 5–10% every year, the cases of sepsis caused by fungi have increased by more than 200% in the US between 1979 and 2000 (Martin et al., 2003). Since both types of pathogens, bacteria and fungi, require fundamentally different anti-microbial therapies, the early classification is crucial. Furthermore, it has been shown that prompt treatment is a prerequisite for successful therapy, as each hour of delay reduces the chances of survival on average by 8% (Kumar et al., 2006). This direct relation emphasizes the necessity for quick and reliable classification methods.

Blood cultures (BCs) and PCR-based assays are currently the standard diagnosis techniques to detect causative pathogens. While BCs aim for the isolation, identification, and susceptibility tests of microorganisms (Westh et al., 2009), molecular pathogen detection by PCR solely enables identification of the pathogen (Schreiber et al., 2013). Numerous studies comparing both methods conclude that the time BCs require to provide positive results is too slow for guiding therapy (Westh et al., 2009; Bloos et al., 2010; Lehmann et al., 2010; Schreiber et al., 2013). Thus, PCR-based assays, which exhibit a turnaround time of several hours may be an important additive tool (Lehmann et al., 2010).

Both methods, BC and PCR, identify the microorganisms directly in the blood. However, at the time of diagnosis, the pathogen may have left the bloodstream, while it still triggers the dysregulated response of the immune system of the host. Thus, another promising approach is to analyze the immunological imprint of the pathogen and infer the pathogen type based on the transcriptional response to the infection. Previous studies have shown that genome-wide transcriptome analysis facilitates the identification of genes with specific expression signatures in sepsis data (Prucha et al., 2004; Shanley et al., 2007). As these genes quantify the state of acute sepsis, they can be considered as biomarkers for this condition. Other research groups used biomarkers to distinguish the microorganisms causing the infection, or to predict the survival chances of infected patients (Pachot et al., 2006; Pankla et al., 2009). Furthermore, septic shock patients have been successfully classified into subgroups using whole-blood gene expression data from microarrays (Wong et al., 2010). Therefore, incorporation of host response transcription data holds great potential to get insights into the systemic host reaction, thus leading to an improved pathogen detection and differentiation. Especially with respect to the rapid increase in incidence of fungal induced sepsis cases, an early detection of fungal sepsis would be of great value.

The genome-wide approach of this study provides an unbiased screening. This strategy facilitates the identification of transcriptional biomarkers featuring distinct expression signatures depending on whether the infectious pathogen is of bacterial or fungal origin. A classifier based on these biomarkers enables the classification of causative microorganisms in new samples. Here, we apply a whole-genome approach for screening the transcriptional response to blood infections and to identify biomarkers. For clinical application, however, a technology like western blot or PCR, which is faster and more accurate or relevant would be advantageous for measuring expression intensities of the biomarker genes. Nevertheless, the present study gives a starting point for the development of a classification device such as a biochip. We based this work on a whole-blood model, as this model takes the *in vivo* complexity of immune responses into account and, compared to other model organisms, the blood components are similar to the human organism with respect to their abundance and functioning (Maccallum, 2012; Hünniger et al., 2014).

## 2. Materials and methods

### 2.1. Microarray data generation and preprocessing

A human whole-blood model was used as described previously (Hünniger et al., 2014). Briefly, HBSS (for mock-infected control) or the human pathogenic fungi *Candida albicans* SC5314 (Gillum et al., 1984) and *Aspergillus fumigatus* ATCC46645 (each 1 × 10^6^/ml), the Gram-positive bacterium *Staphylococcus aureus* ATCC25923 (1 × 10^6^/ml) and the Gram-negative bacterium *Escherichia coli* ATCC25922 (4 × 10^3^/ml) were added to anti-coagulated blood of healthy human donors (male, ≤40 years of age) and incubated at 37°C with gentle rotation for 4 or 8 h. The samples of all pathogens cover three or four different donors with one or two samples each. Infected blood was collected and stored in PAXgene Blood RNA Tubes (PreAnalytiX) to stabilize intracellular RNA until further use. RNA isolation was performed using the PAXgene Blood RNA Kit (PreAnalytiX) corresponding to the manufacturer's instruction. The Illumina°ledR TotalPrep™ RNA Amplification Kit (Ambion) was used for RNA amplification and cRNA transcription. RNA concentrations and quality were assessed by NanoDrop 1000 (Thermo Scientific) and Agilent 2100 Bioanalyzer (Agilent Technologies). Expression levels of RNA samples were analyzed with Illumina°ledR HumanHT-12 v4 Expression BeadChip Kit (Illumina) following manufacturer's protocol. The chip data was background corrected and log-transformed by applying the functions “lumiR” and “lumiT” of the R package “lumi” (Du et al., 2008). Genes with a detection *p* < 0.01 in at least one sample were considered as expressed. Putative and/or not well-characterized genes (i.e., gene symbols starting with ENSG, NT_, LOC, MGC, HS., FLJ, KIAA, or CxORF) were removed, leaving 10449 genes for analysis. The microarray data have been deposited in NCBI's Gene Expression Omnibus (Edgar et al., 2002), accession number GSE65088 (http://www.ncbi.nlm.nih.gov/geo/query/acc.cgi?acc=GSE65088).

### 2.2 Reference genes based normalization

The normalization followed the approach of Vandesompele et al. (2002) which is based on non-normalized expression values of all samples. From a list of putative control genes covering housekeeping genes and reference genes suggested previously by Stamova et al. (2009) and Kwon et al. (2009), genes with most stable expression were selected. First, the gene stability measure *M* as introduced by Vandesompele et al. was calculated for each control gene as the average pairwise variation of a gene, i.e., the pairwise standard deviation of the ratios of the control gene to all other control genes. Thus, genes with lower *M* values are associated with a more stable expression. Iteratively, the gene with the largest *M* value was removed and the calculation was repeated. In this way, a ranking of genes was obtained, representing their stability. The geometric mean of the expression values of the *n* best ranked genes was then used as normalization factor (NF_*n*_)—as a vector for all samples.

Initially, the three most stable genes (NF_3_) were used to determine the optimal number of genes for NF calculation. Then, more genes were successively included (NF_4_, NF_5_, …) as long as the inclusion leads to significant changes on the normalization factor. To quantify these changes, the pairwise variations of each two consecutive NFs were computed. As threshold, 0.15 was used as recommended by Vandesompele et al. A value surpassing this threshold indicate that the inclusion of another gene into calculation is necessary.

### 2.3. Selection of differentially expressed genes

Differentially expressed genes were determined using the Bioconductor package “limma” (Gentleman et al., 2004; Smyth, 2005) of the statistical programming language R. Limma fits linear models to the expression values of each gene and determines differential expression using moderated t-statistics. *P*-values were adjusted according to the method of Benjamini and Hochberg (1995). Genes with an adjusted *p* < 0.05 and a log_2_-fold change of at least ±1 were regarded as differentially expressed.

### 2.4. The random forest classifier

The random forest classifier was built using the “randomForest” package (Liaw and Wiener, 2002) for the R programming language. There are two main parameters which may influence the performance of the classifier: *ntree* and *mtry*. While *ntree* describes the number of trees that are built by the random forest algorithm, *mtry* represents the number of genes used at each split when building a tree. Svetnik et al. (2004) and Díaz-Uriarte and Alvarez de Andrés (2006) showed that the random forest algorithm features high predictive performance, even without parameter adjustment. Only the number of trees needs to be sufficiently large to get stable results. Therefore, the random forest classifier was built growing 100,000 trees. A cross-validation examining the effect of changing *mtry* and *ntree* showed that altering the parameters has no effect on the classification accuracy (Supplementary Material). Thus, we kept the parameter *mtry* on its default value, which is $\left\lfloor \sqrt{g}\right\rfloor$, where *g* is the number of genes of the input dataset.

For the selection of biomarker genes, the measure “mean decrease in accuracy” was used for determining the variable importance values for each gene. The importance values were computed for each class (fungal, bacterial, and mock-infected class) by building random forests with 100,000 trees. The normalized dataset, which was reduced to the data of differentially expressed genes, was used as input.

We scaled the certainty score to a range from 0 to 1. Before scaling, the score represents the proportion of class predictions from all trees of the random forest, which yield the same class as the final classification by the classifier. Let *p* be this proportion and let *N* be the number of possible classes (in this study, *N* = 3, as we consider a fungal, a bacterial, and a mock-infected class), then the certainty score is calculated as

(1) $$certainty\;score=\frac{p-\frac{1}{N}}{1-\frac{1}{N}}.$$

### 2.5. Performance assessment

**Figure 1:**
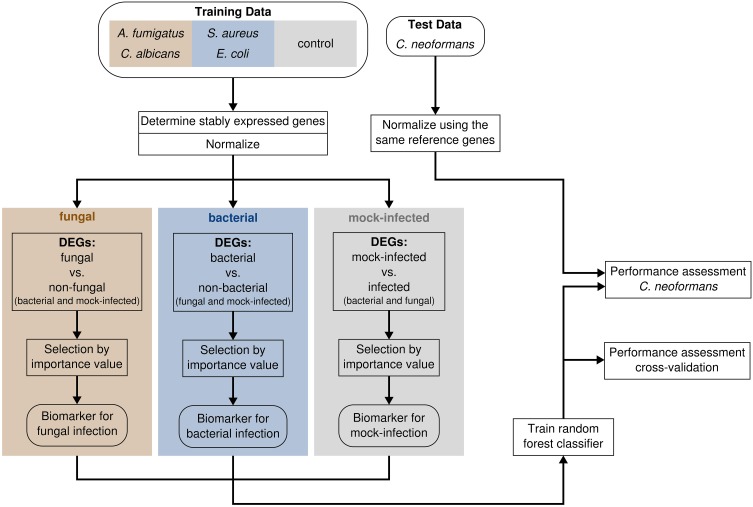
**The workflow for biomarker identification, classifier construction and performance assessment**.

The *C. neoformans* (strain H99, provided by Robin May, University of Birmingham) dataset was generated identical to the other fungi data and quantified using the same chip technology. Expression levels were measured 4 h (3 donors) and 8 h (3 donors) post infection. Mock-infected control samples were simultaneously produced. Before classification, the expression intensities were normalized based on the reference genes which were determined previously without the *C. neoformans* data (Figure 1).

Multidimensional scaling (MDS) was performed using the “cmdscale” function of R. After determining the Spearman correlation of the samples of the normalized *C. neoformans* dataset, the Euclidean distances between these samples were calculated based on the correlation matrix and used as input for the MDS computation. In this way, samples with high correlations are close to each other in the MDS plot.

## 3. Results

### 3.1. Reference genes based normalization

Our first step in building a classifier which discriminates between bacterial and fungal infection is to normalize gene expression values with help of reference genes (Figure 1). The motivation of using reference genes instead of the control samples for normalization is that our classifier should be able to be applied in clinical settings, i.e., for patients, where no control samples exist. To identify reference genes, we used a knowledge driven and data driven approach. First, we considered 10 known housekeeping genes as well as 17 reference genes which were previously suggested by Kwon et al. (2009) and Stamova et al. (2009) (Table 1). Next, we checked which of those genes have a stable expression profile within our dataset. Therefore, we followed the method proposed by Vandesompele et al. (2002), where the stability of a gene is determined on the basis of ratios of raw gene expression values (Materials and Methods). The normalization factor (NF) is then calculated as the geometric mean of the most stably expressed reference genes.

**Table 1:** **Housekeeping genes and putative reference genes suggested by other studies were used as input for determining stably expressed reference genes**.

**Housekeeping genes listed at Vandesompele et al**.	**Reference genes suggested by Stamova et al**.	**Reference genes suggested by Kwon et al**.
*ACTB*	*TRAP1*	*ZNF207*
*B2M*	*DECR1*	*OAZ1*
*GAPDH*	*FPGS*	*LUC7L2*
*HMBS*	*FARP1*	***CTBP1***
*HPRT1*	*MAPRE2*	*TRIM27*
*RPL13A*	*PEX16*	*GPBP1*
*SDHA*	*GINS2*	*ARL8B*
***TBP***	***CRY2***	*UBQLN1*
*UBC*	*CSNK1G2*	*PAPOLA*
*YWHAZ*	*A4GALT*	*CUL1*
		*DIMT1L*
		*FBXW2*
		*SPG21*

*The symbols in the genes *FPGS, FARP1, PEX16, GINS2, A4GALT*, and *SPG21* could not be found in our dataset and thus were not considered. The genes exhibiting the most stable expression are bolded*.

From the 27 considered genes, we determined *CTBP1, TBP*, and *CRY2* as the most stable ones. When comparing the pairwise variations of all successive NFs, we found that using only the three most stably expressed genes is sufficient for producing an accurate NF (Supplementary Figure 2). Including a fourth reference gene leads to no significant changes of the NF, indicated by a low pairwise variation of 0.0496. This value is below the threshold of 0.15 that was recommended by Vandesompele et al. for including more genes. Furthermore, the Spearman correlation between NF_3_ and NF_4_ is >0.99, which also demonstrates that considering a fourth gene is not necessary.

### 3.2. Selection of biomarker genes

The identification of biomarkers, i.e., genes with a specific expression pattern in case of a whole-blood infection, requires the reduction of the gene set by so called feature selection. As gene expression data is high-dimensional by nature, feature selection is one of the most important tasks when building a classifier based on genome wide transcription data. The aim of feature selection is to pick the most informative genes and to remove irrelevant predictors, thus resulting in a dimension reduction. In this way, we can reduce the complexity of the classification while at the same time the predictive performance can be increased. In general, we can distinguish three types of feature selection: filter methods, wrapper methods, and embedded methods (Saeys et al., 2007).

We performed feature selection using the filter and the embedded approach by first determining differentially expressed genes (DEGs) and then selecting genes which are most important for accurate classification (Figure 1). To identify genes showing different expression patterns between the pathogen types rather than between the species, we grouped data into three classes. The fungal species *C. albicans* and *A. fumigatus* form the class “fungal,” while the bacterial species *S. aureus* and *E. coli* were assembled to the “bacterial” class. The samples of the control group are represented by the class “mock-infected.”

#### 3.2.1. Selection of differentially expressed genes

To identify transcriptional responses related to blood infection by fungi or bacteria we determined DEGs for the three classes. A gene is regarded as a DEG for one class, if its expression levels are significantly different to both other classes merged together (Materials and Methods). In this way, we found 204 DEGs for the fungal class, 184 for the bacterial class, and 150 for the mock-infected class. Of these genes, 68 were identified as differentially expressed in all 3 classes simultaneously. The union of the three sets of DEGs comprises a total of 402 genes.

#### 3.2.2. Selection by importance value

**Figure 2:**
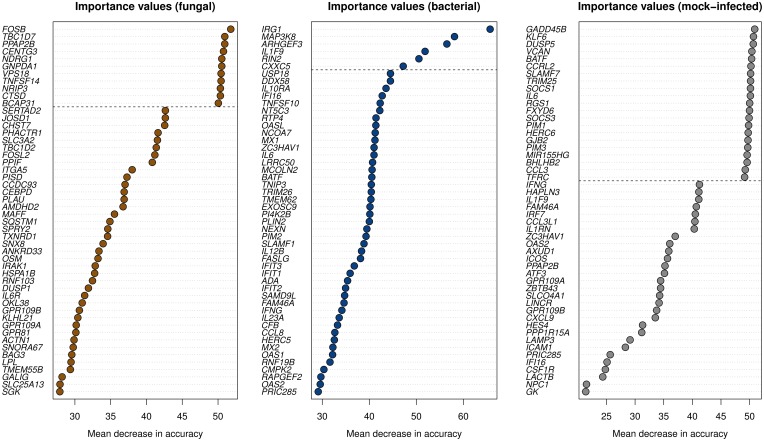
**The variable importance values were computed by the random forest algorithm**. A gene with larger values exhibits a higher influence on the correct class predictions. The 50 highest importance values of the measure “mean decrease in accuracy” are shown. Genes above the dashed lines were selected as biomarkers for the corresponding classes.

We further reduced the set of DEGs to genes being most important for accurate classification. To identify these genes, we used the variable importance measure integrated in the random forest algorithm (Materials and Methods). We selected the top 11, 6, and 21 genes for the classes fungal, bacterial, and mock-infected, respectively, as these genes form groups covering the highest importance values (Figure 2). They are biomarkers for their respective group of pathogens.

#### 3.2.3. Functional annotation of selected biomarker genes

To get insights into the function of the biomarker genes, we performed a Gene Ontology (GO) (Ashburner et al., 2000) enrichment analysis. We employed the tool “GOrilla” (Eden et al., 2009) to identify over-represented GO categories. This web-based tool uses an hypergeometric model to test for enrichment and performs *p*-value adjustment for multiple testing according to the false discovery rate.

At a significance level of 0.05 we found 32 enriched GO terms connected to the identified biomarker genes (Supplementary Table 2). The list comprises terms from the areas of signal transduction, activation of the immune system, response to cytokine stimuli, and down-regulation of phosphorylation. Besides that, GOrilla also identified the category “regulation of sequence-specific DNA binding transcription factor activity” as over-represented. Although numerous of the enriched GO terms are connected to the immune response, we found that multiple biomarkers are related to other processes. For example, genes are involved in cellular growth (*TBC1D7, GADD45B*), vesicle transport (*VPS18*), cell proliferation (*PIM1, PIM3*), cell adhesion (*VCAN*), ion transport (*FXYD6*), or iron uptake (*TFRC*).

Many genes of our biomarkers are already linked to sepsis by other studies. While *IL6* was previously identified as biomarker for sepsis (Pierrakos and Vincent, 2010), *GADD45B, SOCS3*, and *IRG1* were shown to be up-regulated in septic patients (Johnson et al., 2007; Li et al., 2013). Moreover, it has been shown that *IL1F9* is up-regulated by *S. aureus* cell wall proteins in human peripheral blood mononuclear cells (Kang et al., 2012). Furthermore, *RGS1, CCL3*, and *SOCS1* were connected to sepsis in animal studies (Panetta et al., 1999; Takahashi et al., 2002; Grutkoski et al., 2003), while for *CTSD* increased expression levels were observed in mice with induced septic shock (Yoo et al., 2013). *MAP3K8* is linked to sepsis in mice, with being crucial for the TNF production (Mielke et al., 2009). Furthermore, the gene *MIR155HG* showed significantly higher expression values in samples with bacterial or fungal infection than in the mock-infected controls. This gene encodes for the microRNA miR-155, which is known to be involved in the regulation of antimicrobial immune response (O'Connell et al., 2007; Rodriguez et al., 2007; Das Gupta et al., 2014).

**Figure 3:**
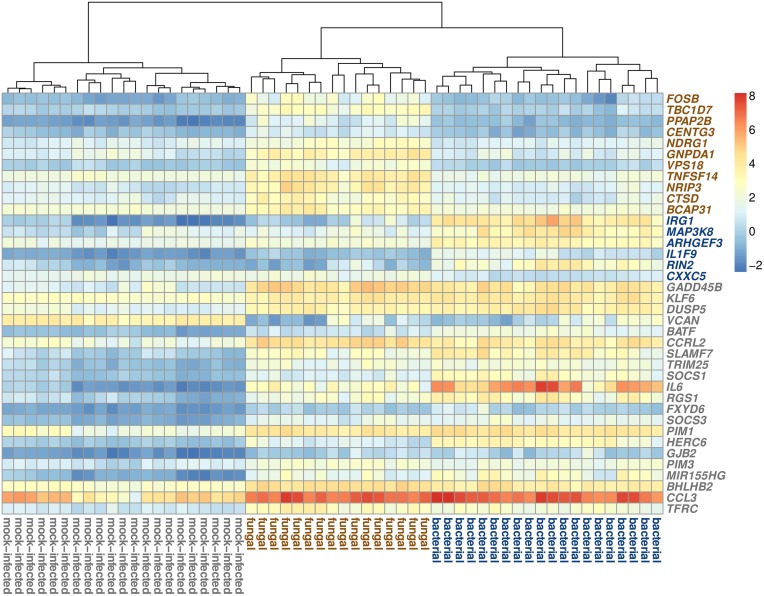
**Visualization of the expression patterns of the biomarker genes**. The samples are clustered according to their corresponding classes. The heatmap colors correlate with the normalized expression intensities (see key on right side). The colors of the gene symbols indicate the class for which the gene was selected as biomarker (brown = fungal class, blue = bacterial class, gray = mock-infected class).

Examining the expression signatures of the selected genes (Figure 3, Supplementary Figure 1), we discovered that for the fungal and bacterial class, most genes are up-regulated, compared to the respective other two classes. Of the six biomarkers for bacterial blood infection, only one gene (*CXXC5*) was down-regulated, while the other five genes showed up-regulation. For the fungal class, all 11 selected genes were up-regulated. We observed different patterns for the genes of the mock-infected class. Twenty of the 21 genes were down-regulated in the control samples and one gene (*VCAN*) was up-regulated.

Taken together, our feature selection approach was able to identify biomarker genes, which have been shown to be involved in sepsis and also cover a broad range of biological processes.

### 3.3. Building the classifier

To determine if an infecting pathogen of an unknown whole-blood sample is of fungal or bacterial origin, the sample is classified using the expression data of the selected biomarkers. We accomplish the classification by a random forest (Breiman, 2001) classifier (the classifier can be found as R object as supplementary file). Random forest is based on an ensemble of decision trees, where each tree is built on a different random subset of the input data. The output of the classifier is determined by the majority vote of the class predictions of all trees. As we used 100,000 trees, the algorithm provides us with 100,000 single classifications. We utilized the votes of the trees to introduce a certainty score for the final classification. This score represents the fraction of class predictions identical with the final classification and was scaled to a range from 0 to 1 (Materials and Methods). In case of a certainty score of 1, all trees have predicted the same class for a given sample and consequently this class was then output by the classifier. On the other hand, the certainty score is 0, if all tree votes are equally distributed across all possible classes. Thus, the score indicates, how sure the classifier is about its decision. Calculating the certainty score for the training data, we achieved average values of 0.941, 0.966, and 0.99 for fungal, bacterial, and mock-infected class, respectively.

### 3.4. Performance assessment

Having built our classifier, we next studied its performance in distinguishing between fungal or bacterial blood infection. Our aim was to accurately classify new samples by the given classification model. Therefore, the performance assessment methods have to yield unbiased accuracy rates. To get unbiased estimates of accuracy, the samples for testing the classifier should be independent from the samples for training the classifier. We fulfilled this requirement with additionally independently created data comprising RNA expression measurements of human whole-blood samples infected with *C. neoformans*. An additional approach to assess a classifiers performance is cross-validation. Cross-validation emulates independent test sets in an iterative technique and in this way resolves the need for true test data. Furthermore, we evaluate the ability of the classifier to handle fluctuations in the expression values by classifying samples after adding random noise to the data (Supplementary Material).

#### 3.4.1. Test data of *C. neoformans*

To assess the performance of the classifier on an independent test set, we created a new dataset of RNA expression measurements of human whole-blood infected with *C. neoformans*. The data comprises 6 samples of fungal infection and 6 mock-infected controls (Materials and Methods). Being part of the phylum of Basidiomycota, *C. neoformans* is a phylogenetically and morphologically very different fungus compared to *C. albicans* and *A. fumigatus*, both belonging to the phylum of Ascomycota (James et al., 2006).

**Figure 4:**
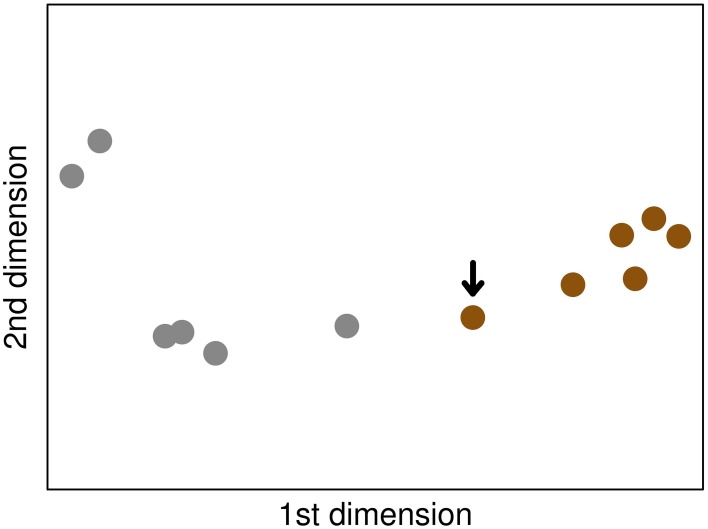
**The MDS plot based on the *C. neoformans* dataset, where the relative positions in the plot represent the Euclidean distances of the Spearman correlations of the samples**. Small distances correspond to high correlation coefficiens. Brown and gray circles indicate samples of the fungal and the mock-infected class, respectively. The arrow marks the fungal sample that was misclassified as mock-infected control.

When assessing the classification performance using the new data, our model correctly classified 5 of the 6 fungal samples (83.3%). One sample was wrongly classified as mock-infected. All classifications of the mock-infected samples were performed correctly. In this way, we achieved an overall accuracy rate of 91.7%. The sensitivities are 83.3 and 100%, while the specificities are 100 and 83.3% for fungal and mock-infected class, respectively (Table 2). We examined the misclassification in more detail by a correlation analysis using a multidimensional scaling (MDS) plot (Figure 4). MDS is a dimension reduction technique, producing an easy-to-visualize output showing relationships within the data. The plot revealed that the misclassified sample shows more similarity to the data of mock-infected class than the other *C. neoformans* samples.

**Table 2:** **Sensitivities and specificities for the performance assessments**.

	**Sensitivity**			**Specificity**		
	**Bacterial**	**Fungal**	**Mock-infected**	**Bacterial**	**Fungal**	**Mock-infected**
*C. neoformans* predictions	–	0.833	1.000	–	1.000	0.833
Cross-validation	0.950	0.938	1.000	0.973	0.976	1.000

*The C. neoformans dataset does not comprise samples of the bacterial class. Thus, no sensitivity and specificity could be calculated for this condition*.

The difference in the accuracy values between the two classes is also reflected in the certainty scores. We obtained an average certainty of 0.475 (± 0.190) for all fungal samples, whereas for the mock-infected samples we achieved an average score of 0.810 (± 0.165). When splitting the fungal specimen into falsely and correctly classified ones, the observed certainty value for the misclassified sample is higher, 0.654, than for the right classifications, 0.439.

#### 3.4.2. Cross-validation

When the sample size of a study is relatively small, it is preferred to use all available samples in feature selection and training. However, this leads to a lack of test data. Cross-validation is a widely used method to overcome this problem by emulating independent test sets without using additional datasets. It works by iteratively setting aside samples for testing, while the remaining samples are used to train the model. The split is performed in the way that each sample of the data is exactly once in the test set. In this way, cross-validation guards against overfitting.

To estimate how accurate the classifier will perform on independent data, we carried out a stratified 10-fold cross-validation (CV). It is important that CV encompasses all feature selection steps, as otherwise a selection bias is induced (Ambroise and McLachlan, 2002). Therefore, we conducted the following procedures on the training set in each CV iteration: determine DEGs, rank the DEGs according to their importance value, select the top-scoring genes, and train a random forest classifier.

In compliance with the CV procedure, the class of each sample of our dataset was predicted and the accuracy of the classification model was estimated. Of the 57 samples, only two were misclassified, while 55 classifications were correct. The two wrong classifications appeared for one bacterial and one fungal sample. All data of the mock-infected class was classified correctly. Thus, the average accuracy of the CV is 96.49% (sensitivities: 93.8, 95, 100% for fungal, bacterial, and mock-infected class; specificities: 97.6, 97.3, 100% for fungal, bacterial, and mock-infected class; Table 2). The average certainties of the classifications were 0.795 (± 0.169), 0.855 (± 0.18), and 0.937 (± 0.085) for the classes fungal, bacterial, and mock-infected, respectively.

## 4. Discussion

Here we present an transcriptome analysis of human whole-blood data comparing bacterial and fungal infections with mock-infected control samples. Based on the regulatory differences, we identified biomarker genes, which show characteristic expression patterns according to their respective causative pathogen type. The selection was not only based on statistical significance. It also took into account to what extent the random forest classification algorithm assesses these genes as important for separating the given classes. In this way, we applied two different methods of feature selection: the filter approach and the embedded approach. With the detection of differentially expressed genes we are able to remove most of the irrelevant genes and extract a set of potential transcriptional marker genes. The selection by differential expression is a widely used method for identifying sepsis related marker genes (Prucha et al., 2004; Pachot et al., 2006; Shanley et al., 2007; Pankla et al., 2009). The subsequent calculation of gene importance values using the random forest algorithm allows us to identify the genes showing the strongest and most constant up- or down-regulation as a consequence of the blood infection by the particular type of microorganisms. In this way, we were able to remarkably reduce a set of whole-genome expression measurements to significant signatures distinguishing bacterial from fungal infections and mock-infected controls. The genes identified as biomarkers for the mock-infected class exhibit similar signatures for both infection types, fungal and bacterial. Most of these genes show down-regulation in the mock-infected samples. However, at the same time they were up-regulated in the infected samples, irrespective of the infecting pathogen type. Therefore, they possibly reflect cellular regulations to respond microbial infections in general. Thus, they can be considered as pathogen-independent markers for whole-blood infections. Studies investigating a broader range of pathogens should be carried out to confirm this hypothesis.

Using a human whole-blood model in this work is supported by several advantages. First, as opposed to purified human immune cells, it also considers the *in vivo* complexity of the immune response in blood (Hünniger et al., 2014). Next, there are no differences in proportions and functioning of the peripheral blood components between this model and the target organism, the human, in contrast to other model organisms like mice (Maccallum, 2012). Furthermore, human whole-blood infection models have been successfully used previously to identify factors of virulence (Echenique-Rivera et al., 2011) and to analyze human immune responses (Tena et al., 2003).

Following a genome-wide approach allows us to consider all genes as potential biomarkers for pathogen type recognition, even if they are not related to immune response. Therefore, with respect to the screening for biomarkers, using a whole-genome method is more promising than techniques which are limited to a small number of candidates, like serum cytokine analysis. Indeed, the selected biomarker genes cover a broad range of functions. In this way, these genes may facilitate the recognition of bloodstream infections even when the immune system of the patient is affected by additional diseases. Besides that, we found the gene *MIR155HG* as up-regulated in the samples with infections. Recently, Das Gupta et al. (2014) have shown that miR-155 up-regulation is not specific to host response on bacterial pathogens. They also detected increased expression levels as reaction to *A. fumigatus* infections. As we observed up-regulations for all considered species, fungi as well as bacteria, our results confirm the findings that miR-155 is involved in a general host response to infections, covering a wide range of pathogens. Besides, numerous of the selected biomarkers were previously associated to sepsis in either human or animal studies. This finding indicates, that although our results are based on an experimental model instead of patient data, we could identify characteristic gene regulations in response to microbial bloodstream infections.

Preceding the feature selection steps, we successfully identified the three most stable genes from a set of published control genes and used them as reference for normalizing the dataset. In this way, we do not use absolute gene expression values to train our classifier. Instead, we use expression values relative to the geometric mean of the reference genes. Regarding the application case, a user of the classifier aims to identify the pathogen type using only a single blood sample without mock-infected controls for comparison. It is well known that the intensity values on microarrays are influenced by technical variations and errors connected with wet lab hand handling of samples as well as hybridization and scanning of the chip. These differences can not be detected on a single sample, but they do affect the absolute intensity values. With normalizing relative to reference genes, we control for this effect, as all genes on the chip are influenced in the same way. Furthermore, this method can easily be adapted to other quantification methods like PCR.

Using the biomarker genes, we trained a random forest classifier to classify the pathogen type in whole-blood samples. Random forest provides several advantages making it suitable for this study. It is fast in training and testing, supports multiclass classifications and provides the variable importance for evaluating the input features. With this embedded measure, we were able to select the best class-separating genes leading to a small set of biomarkers. There are further classification methods like support vector machines or naïve Bayes classifiers, which were successfully applied on microarray data in other studies (Kelemen et al., 2003; Howrylak et al., 2009). For comparison, we tested the classification performance of these two techniques on both the *C. neoformans* dataset and the cross-validation, using the previously selected biomarkers (Supplementary Material). The support vector machine as well as the naïve Bayes method yielded the same classifications of all samples as the random forest model. The fact that the three classification methods are very different in their functional principles and the results are unaffected by the choice of the model indicates that the selected biomarker genes are robust.

The certainty score based on the votes of the trees provides an easy-to-compare measure for assessing the classification quality. It directly reflects the ability of the classification model to properly classify the input data. This means, a class prediction with a high certainty score is more likely to be correct, than one with a low score. One possible application case for this measure is the introduction of a threshold, followed by the removal of low-scoring classifications.

We tested the classifier with an additional dataset comprising whole-blood samples of fungal infection and mock-infected controls. The medically important fungus used for these additional samples, *C. neoformans*, is phylogenetically very different from *C. albicans* and *A. fumigatus*. These differences can lead to varieties in the transcriptional response of the host. However, the accuracy value of about 92% indicate that the selected biomarker genes are largely unaffected. Therefore, these genes are general indicators for whole-blood infections caused by fungi. The MDS analysis revealed that the misclassified fungal sample shows a greater similarity to the specimen of the mock-infected class than to the fungal cases. Although the divergence with the other fungal samples is only small, the differences are sufficient for wrong classification. Consequently, the correct classifications of the *C. neoformans* samples are possibly unsure. Indeed, the certainty values are much lower for the fungal class, compared to the mock-infected controls. Furthermore, we were surprised to find the certainty score of the misclassified sample being higher than the average score of the remaining fungal specimen. This observation confirms the assumption that the prediction of *C. neoformans* as fungal infected blood sample is a difficult task for the classifier, but still leads to mostly correct results.

High accuracy values were not only achieved when validating the classifier with the additional *C. neoformans* dataset, but also when testing it with stratified 10-fold CV. This broadly used performance assessment technique iteratively estimates the accuracy of a prediction model without an independent dataset. The two misclassifications in this test appeared for fungal and bacterial class. The predictions of the fungal and the bacterial class also exhibit the lowest values and the largest fluctuations of the certainty scores. However, it should be noted that the average scores are still high, as 0.795 is the smallest of them.

In summary, the results of the assessments by using an additional dataset of fungal infection, i.e., the external validation, as well as by performing a CV, i.e., the internal validation, are promising. Most of the tested samples were correctly classified, although in some cases right classifications were accompanied by low certainty scores.

We also performed a noise-robustness test to examine whether the classifier can compansate fluctuations in the expression data. The high accuracy rates indicate that the indentified biomarkers are robust with respect to changes in their expression intensities. This robustness is important for a potential clinical application, where patients are of different age, sex, medication, and health condition and thus expression intensities of the same genes will vary between these patients.

The experimental model of this work comprises the infection of blood from healthy human donors with typical sepsis causing microorganisms. Although we gained important insights into the transcriptional response on the pathogens, our findings possibly can not be directly utilized for clinical application. To achieve that, further analyses on gene expression data from septic patients as well as functional follow-up studies have to be performed. Unfortunately, whole-genome expression data from septic patients where the causing pathogen is known is rare in publicly accessible databases. Especially, datasets comprising the transcriptional response to fungal induced sepsis are scarce. Thus, we lack the basis for more clinical relevant investigations, which is why it remains an open task for future research. Furthermore, it should be noted that the presented classifier can not be used to identify the infecting species. Rather it is supposed to answer the question if the pathogen is of bacterial or fungal origin and whether or not it is necessary to administer antimycotics instead of antibiotics. To initiate a species dependent therapy, more requirements have to be fulfilled, e.g., in case of a bacterial infection, the appropriate antibiotic has to be determined by an antibiogram.

In this study we present an effective selection of genes showing characteristic expression patterns depending on the type of the infectious organism. The resulting small gene set was used to train a fast and accurate random forest classifier, which performs well in predicting the class of the pathogen. Examining the transcriptional footprint of the sepsis causing microorganism in the blood of the host is a promising approach for quick pathogen identification. With the presented classification model we meet the increasing challenge of fungal induced septic infections requiring novel detection methods.

## Author contributions

AD did the bioinformatic analysis and co-wrote the manuscript. KH performed the experiments, generated the data, and co-wrote the manuscript. MW discussed the analysis and co-wrote the manuscript. RG, OK, and JL designed the research and co-wrote the manuscript.

### Conflict of interest statement

The Associate Editor, Tunahan Cakir, declares that, despite collaborating on the Frontiers Research Topic “Endothelial cell dysfunction in pathogen-induced hemorrhagic fevers” with the author Reinhard Guthke, the review process was handled objectively and no conflict of interest exists. The authors declare that the research was conducted in the absence of any commercial or financial relationships that could be construed as a potential conflict of interest.
