# Supplementary material for: Biomarker-based classification of bacterial and fungal whole-blood infections in a genome-wide expression study
Source: Front Microbiol. 2015 Mar 11;6:171. doi: 10.3389/fmicb.2015.00171 (PMC4356159; doi:10.3389/fmicb.2015.00171)
Supplement: Supplementary file 1 [file SupplementaryMaterial.pdf]

Supplementary Material: Biomarker-based classification of bacterial and fungal whole-blood infections in a genome-wide expression study

Andreas Dix [1], **Kerstin Hünniger** [2], **Michael Weber** [2], **Reinhard Guthke** [1], **Oliver Kurzai** [2], **and Jörg Linde** [1,*]

[1] *Leibniz Institute for Natural Product Research and Infection Biology Hans-Knöll-Institute, Jena, Germany*
[2] *Septomics Research Centre, Friedrich Schiller University and Leibniz Institute for Natural Product Research and Infection Biology Hans-Knöll-Institute, Jena, Germany*

Correspondence*:
Jörg Linde
Leibniz Institute for Natural Product Research and Infection Biology – Hans-Knöll-Institute, Beutenbergstraße 11a, Jena, 07745, Germany, joerg.linde@hki-jena.de

1 THE RANDOM FOREST CLASSIFIER

We examined the effect of changing the parameters *mtry* and *ntree* on the classification accuracy using a 10-fold stratified cross-validation. As suggested by Liaw and Wiener (**Liaw and Wiener**, 2002), it should be tested if doubling or halving of the default value of *mtry* ($\lfloor\sqrt{g}\rfloor$, where g is the number of genes of the input dataset) has an effect on the results. Furthermore, we reduced the number of trees to 10,000 and 1,000. We found that for all tested values, our results were identical (**Supplementary Table 1**). Additionally, we assessed the performance of the classifier choosing extremely low values for the parameters (*mtry**0.01, *ntree*=10). Due to the small *mtry*, only one gene is considered at each split when building a tree. It could be expected that the accuracies decrease for these settings. However, the calculated accuracy values were unchanged, except for one run using *mtry**2 and *ntree*=10 (**Supplementary Table 1**). The reason for the stability of the results is the strong expression differences between the classes for most of the biomarkers. When examining the expression intensities (**Supplementary Figure 1**), we found that there is nearly no overlap between the expression values of the samples associated to the class of a biomarker gene and the values of the samples of the other two classes. This means, when the random forest algorithm builds a tree based on only two genes from different classes, then a sample can be classified correctly. For example, using a fungal biomarker gene (e.g., *FOSB*) at one split and a bacterial biomarker (e.g., *IRG1*) at another split is sufficient to distinguish between any of the three classes. Moreover, there are cases, where only one gene might be sufficient for classification, as the expression values of this gene are on a different level for each class, e.g., *PPAP2B* or *HERC6* (**Supplementary Figure 1**). As the large expression differences can be found for most selected biomarkers of each class, the classifications yield high accuracies even for very small parameter values. Thus, the parameter *mtry* as well as the number of trees have no major influence on the performance of the classifier.

Supplementary Table 1. The accuracies for the cross-validations using different parameter settings for *mtry* and *ntree*.

	**mtry * 0.01**	**mtry * 0.5**	**mtry * 1**	**mtry * 2**
**ntree = 10**	0.9649	0.9649	0.9649	0.8947
**ntree = 1000**	0.9649	0.9649	0.9649	0.9649
**ntree = 10000**	0.9649	0.9649	0.9649	0.9649
**ntree = 100000**	0.9649	0.9649	0.9649	0.9649

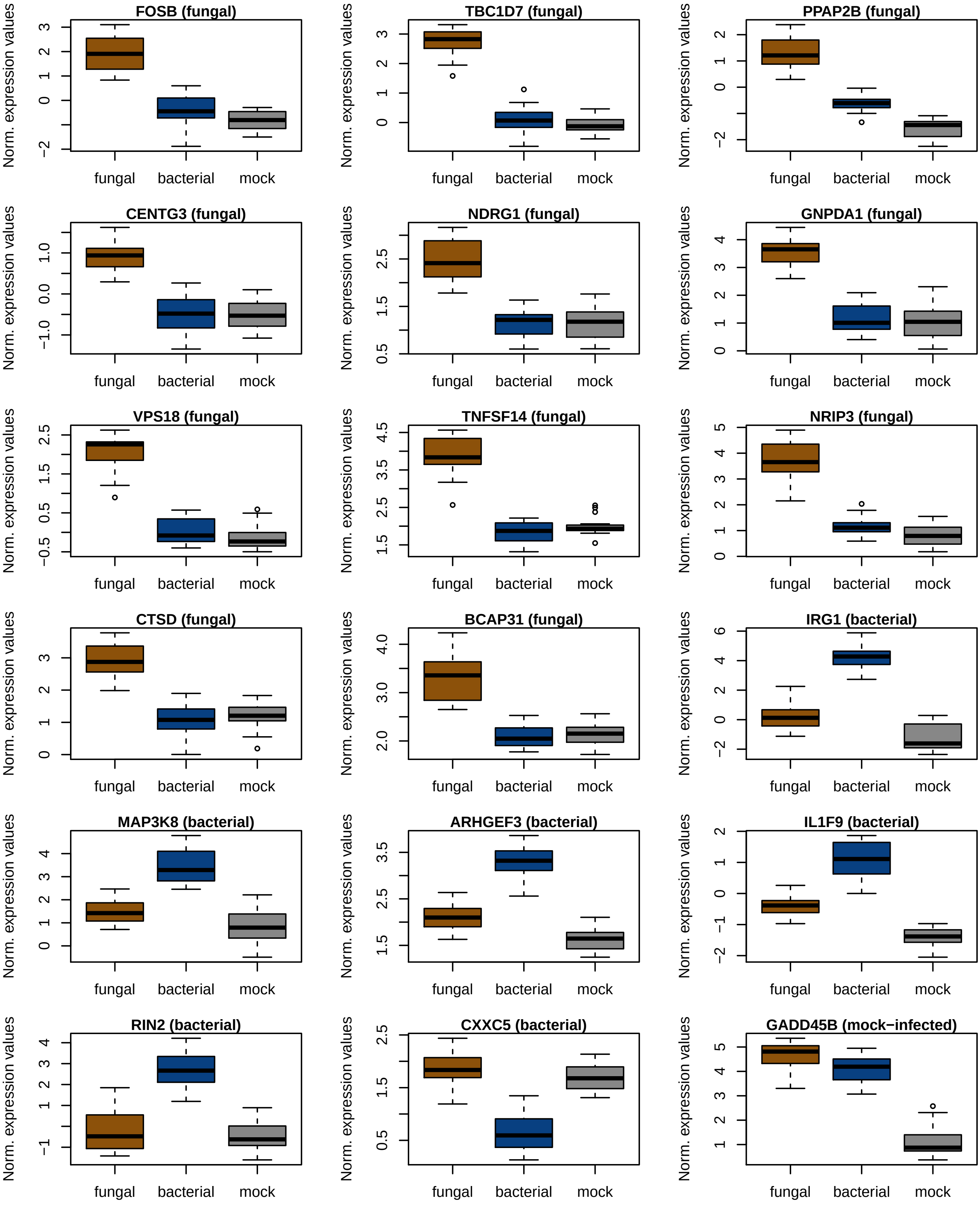

Supplementary Figure 1. Continued on next page.

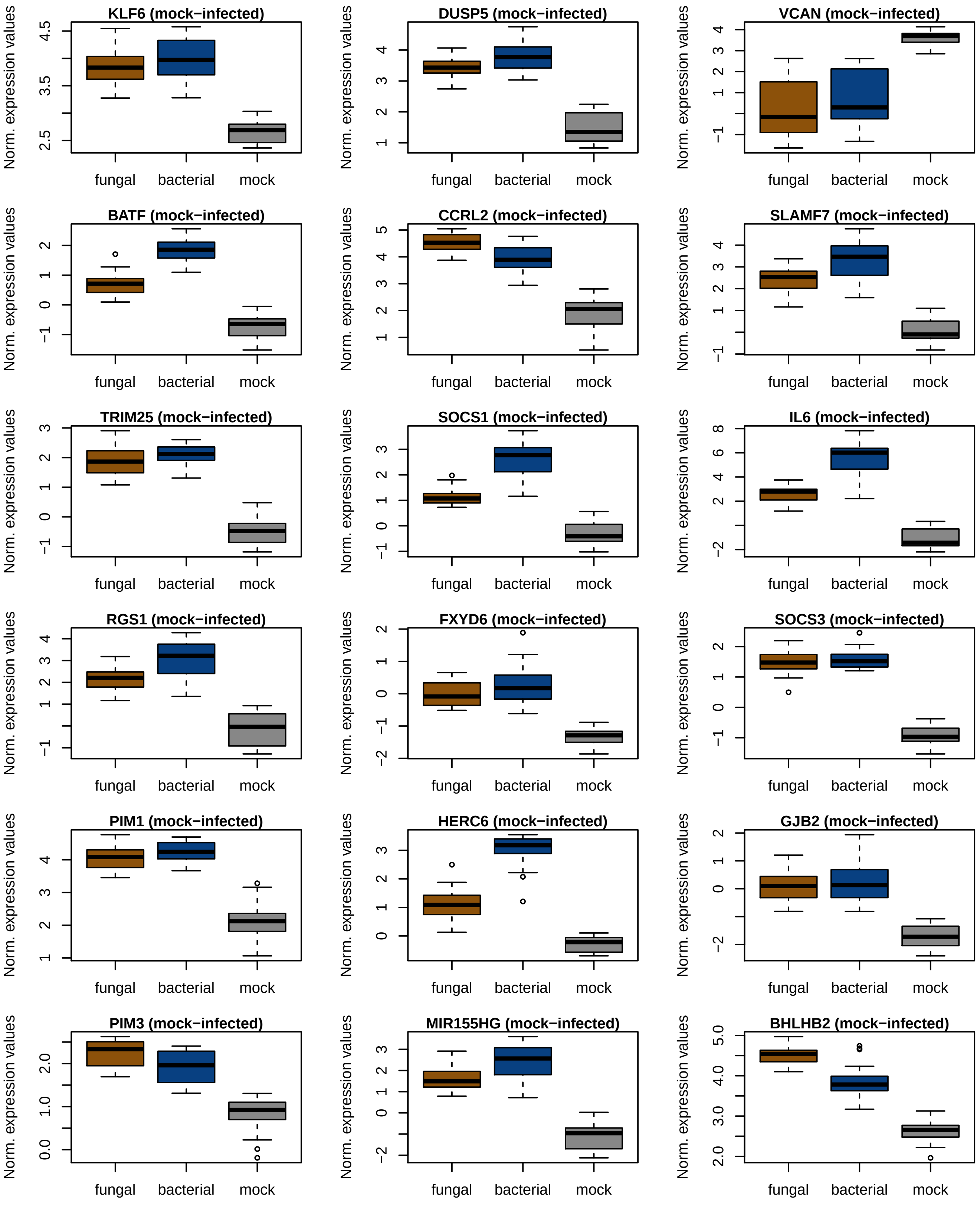

Supplementary Figure 1. Continued on next page.

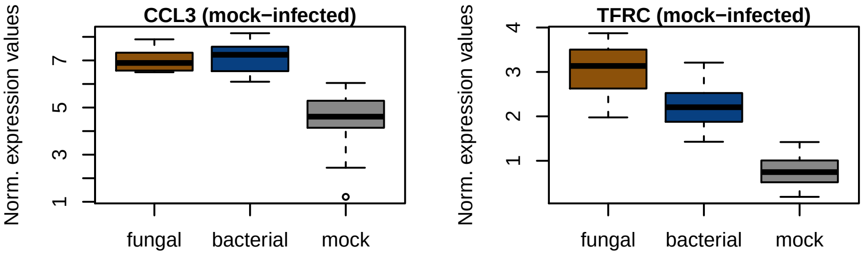

Supplementary Figure 1. Boxplots showing the expression intensities of the biomarker genes. The boxes represent the samples of classes fungal (brown), bacterial (blue), and mock-infected (gray). The class for which a gene was identified as biomarker is indicated in parentheses next to the gene name.

2 REFERENCE GENES BASED NORMALIZATION

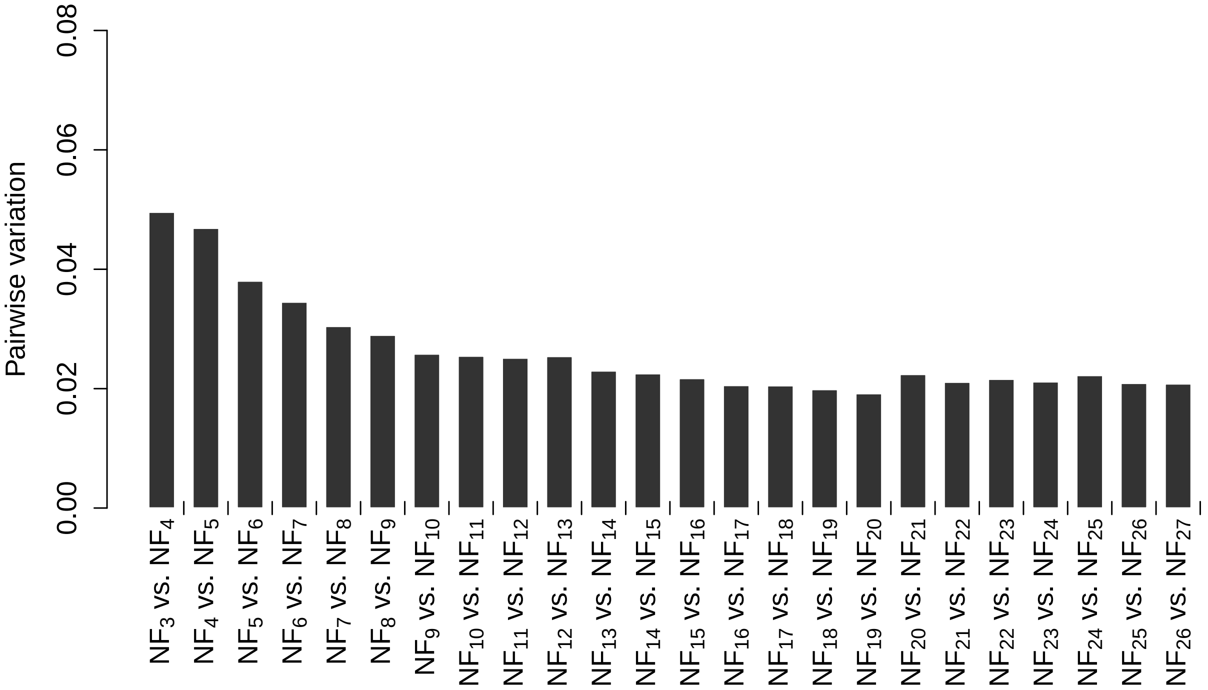

Supplementary Figure 2. The pairwise variations of NF_i and NF_{i+1}, where NF_i denotes the NF based on the i most stable genes. The higher the values, the larger the effect of including another gene on the NF. None of the pairwise variations surpass the threshold of 0.15 for further gene inclusion.

3 SELECTION OF BIOMARKER GENES

Supplementary Table 2. Gene Ontology terms that were connected to the biomarker genes. The list was created using GOrilla and comprises only over-represented terms at an FDR adjusted significance level of 0.05.

GO term	Description	P-value	FDR q-value
GO:0065009	regulation of molecular function	6,78E-009	8,28E-005
GO:0050790	regulation of catalytic activity	7,66E-007	4,68E-003
GO:0023051	regulation of signaling	1,01E-006	4,13E-003
GO:0010646	regulation of cell communication	1,09E-006	3,32E-003
GO:0009966	regulation of signal transduction	1,13E-006	2,76E-003
GO:0019220	regulation of phosphate metabolic process	1,50E-006	3,06E-003
GO:0051174	regulation of phosphorus metabolic process	1,66E-006	2,90E-003
GO:0002376	immune system process	2,18E-006	3,32E-003
GO:1902531	regulation of intracellular signal transduction	2,73E-006	3,71E-003
GO:0001933	negative regulation of protein phosphorylation	8,38E-006	1,02E-002
GO:0048583	regulation of response to stimulus	9,96E-006	1,11E-002
GO:0072540	T-helper 17 cell lineage commitment	1,29E-005	1,31E-002
GO:0042326	negative regulation of phosphorylation	2,38E-005	2,23E-002
GO:0006469	negative regulation of protein kinase activity	2,86E-005	2,50E-002
GO:0071345	cellular response to cytokine stimulus	2,97E-005	2,42E-002
GO:0044092	negative regulation of molecular function	3,12E-005	2,38E-002
GO:0071346	cellular response to interferon-gamma	3,41E-005	2,45E-002
GO:0050896	response to stimulus	3,78E-005	2,57E-002
GO:0033673	negative regulation of kinase activity	3,95E-005	2,54E-002
GO:0051348	negative regulation of transferase activity	5,34E-005	3,26E-002
GO:0010563	negative regulation of phosphorus metabolic process	5,85E-005	3,40E-002
GO:0045936	negative regulation of phosphate metabolic process	5,85E-005	3,25E-002
GO:0043373	CD4-positive, alpha-beta T cell lineage commitment	6,43E-005	3,42E-002
GO:0002295	T-helper cell lineage commitment	6,43E-005	3,27E-002
GO:0034341	response to interferon-gamma	7,61E-005	3,72E-002
GO:0045321	leukocyte activation	8,11E-005	3,81E-002
GO:0051090	regulation of sequence-specific DNA binding transcription factor activity	8,23E-005	3,73E-002
GO:0002363	alpha-beta T cell lineage commitment	8,99E-005	3,92E-002
GO:0034097	response to cytokine	1,04E-004	4,40E-002
GO:0031400	negative regulation of protein modification process	1,13E-004	4,62E-002
GO:0043369	CD4-positive or CD8-positive, alpha-beta T cell lineage commitment	1,20E-004	4,72E-002
GO:0044093	positive regulation of molecular function	1,32E-004	4,88E-002

4 PERFORMANCE ASSESSMENT

4.1 TEST FOR NOISE-ROBUSTNESS

Additionally to the assessment how well our classifier performs on new and/or independent data, we also evaluated its ability to overcome fluctuations in the expression data. We simulated different expression intensities for the selected biomarker genes across all samples by adding increasing levels of noise to the gene expression data (Materials and Methods). Due to this noise, a wider range of intensities is covered by the expression values and the single data points are more and more scattered within this range.

To test for noise-robustness, we simulated noise for the gene expression data. The noise values, which were added to the expression intensities, were generated by producing normal distributed random values at mean 0 and standard deviation σ. The magnitude of σ was based on the average standard deviation (ASD) of all genes in the analysis, which was 0.4859. Then the ASD was multiplied with scalars (0, 0.5, 1, 1.5, 2, 2.5, 3, 3.5, 4, 4.5, 5) to increase the effect stepwise. The noise was included before normalizing the data.

The noise effect was included on the raw data, so that the data processing steps (i.e., normalization and classification) are applied on the manipulated data. We calculated 11 levels of increasing noise, based of the ASD of all genes. For each of these levels, we repeated for 1000 times the process of drawing a random sample, adding noise, normalizing the gene expression values by the reference genes, and classifying the sample according to the type of infection. The accuracy decreases for increasing amounts of noise (see **Supplementary Table 3** for sensitivities and specificities). For up to 2*ASD, over 95% of the classifications were still correct, while we achieved accuracies of 88%, 78%, and 74% for 3, 4, and 5 times ASD, respectively (**Supplementary Figure 3**).

Similarly to the accuracy, the certainty scores are dropping with increasing noise (**Supplementary Figure 4**). Starting at 0.975, the average score is decreasing to 0.352 for 5*ASD. However, unlike the accuracy rates, the decrease is large between lower noise levels and eases for higher amounts of noise. Separating the certainty scores according to the classes revealed that the mock-infected samples achieved the highest scores. Classification of the fungal and bacterial samples, however, led to lower certainty scores, while the values of both classes were rather similar.

Supplementary Table 3. Sensitivities and specificities for the levels of increasing noise.

	**Sensitivity**			**Specificity**		
	bacterial	fungal	mock-infected	bacterial	fungal	mock-infected
0.0*ASD	1.000	1.000	1.000	1.000	1.000	1.000
0.5*ASD	1.000	1.000	1.000	1.000	1.000	1.000
1.0*ASD	0.997	0.985	0.995	0.997	0.997	0.995
1.5*ASD	0.980	0.971	0.981	0.998	0.983	0.986
2.0*ASD	0.964	0.922	0.983	0.995	0.986	0.956
2.5*ASD	0.923	0.888	0.930	0.991	0.954	0.927
3.0*ASD	0.862	0.843	0.933	0.992	0.940	0.890
3.5*ASD	0.782	0.752	0.863	0.982	0.906	0.814
4.0*ASD	0.758	0.707	0.857	0.969	0.904	0.796
4.5*ASD	0.732	0.726	0.836	0.970	0.871	0.813
5.0*ASD	0.724	0.663	0.825	0.964	0.874	0.777

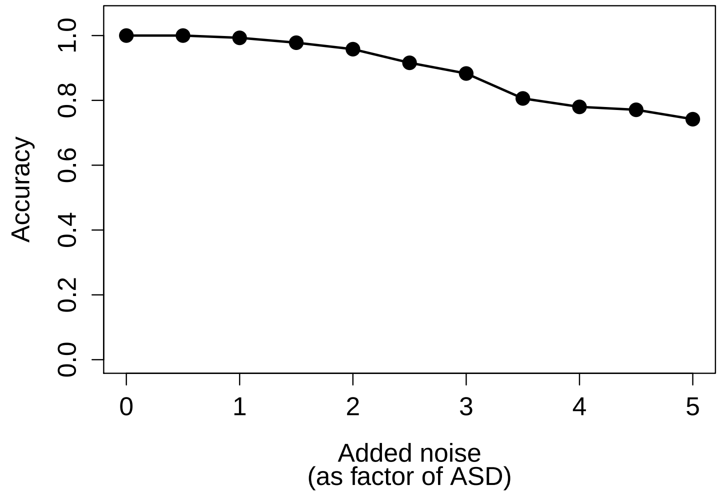

Supplementary Figure 3. The accuracy values as fraction of correct predictions for increasing noise levels. The ASD was multiplied with increasing factors from 0 to 5. The product was used to simulate incremental rising noise of the dataset.

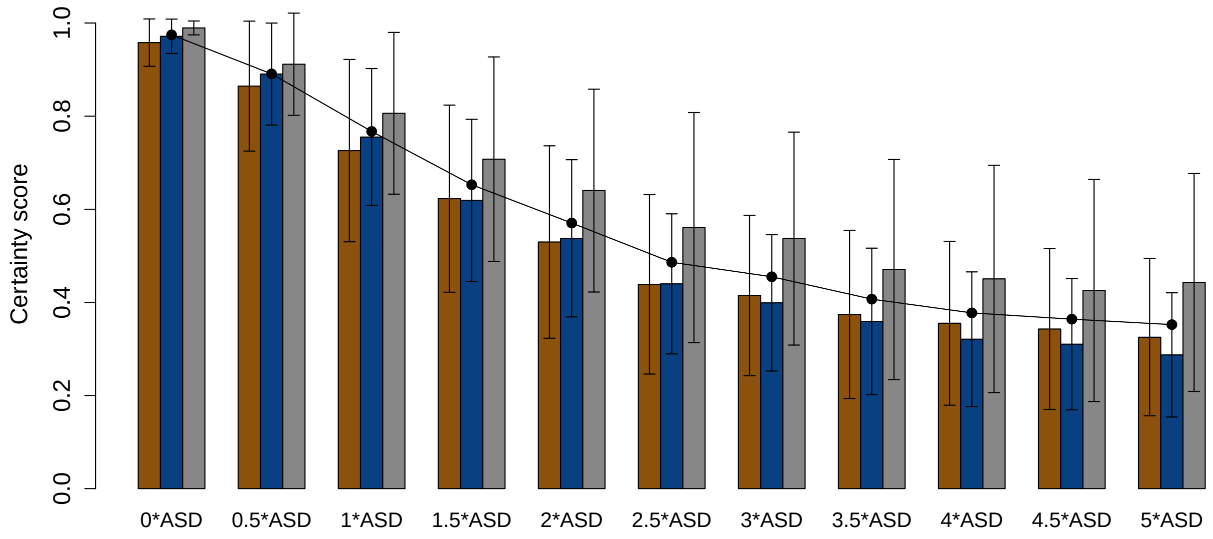

Supplementary Figure 4. The certainty scores of the predictions were calculated in dependence on the noise level. The bars in brown, blue, and gray represent the average scores of fungal, bacterial, and mock-infected samples, respectively. The whiskers indicate the standard deviations. The solid line marks the average certainty scores of all three classes together. Across all noise levels, the mock-infected class yielded the highest certainty values. For all classes, the highest decreases were observed between lower noise values.

5 DISCUSSION

The support vector machine was created using the R-package "e1071" (**Meyer et al.**, 2012). The data was internally scaled to zero mean and unit variance. We used the default parameters of the "svm" function (radial basis function kernel, γ = (1 / data dimension), cost C = 1). The naïve Bayes classifier was built with the function "naiveBayes" from the "e1071" package. This function assumes that the features are independend and follow a Gaussian distribution. We calculated the confusion matrices of the classifications using cross-validation and the *C. neoformans* dataset, respectively, with application of random forest, support vector machine, and naïve Bayes classifier (**Supplementary Tables 4 and 5**).

Supplementary Table 4. The confusion matrices of the cross-validation using the classification methods random forest, support vector machine, and naïve Bayes classifier. The classifications of the samples are identical for all three methods.

Random forest

		**Predicted class**		
		bacterial	fungal	mock-infected
**True class**	bacterial	19	1	0
	fungal	1	15	0
	mock-infected	0	0	21

Accuracy: 0.9649

Support vector machine

		**Predicted class**		
		bacterial	fungal	mock-infected
**True class**	bacterial	19	1	0
	fungal	1	15	0
	mock-infected	0	0	21

Accuracy: 0.9649

Naïve Bayes classifier

		**Predicted class**		
		bacterial	fungal	mock-infected
**True class**	bacterial	19	1	0
	fungal	1	15	0
	mock-infected	0	0	21

Accuracy: 0.9649

Supplementary Table 5. The confusion matrices of the classification results of the *C. neoformans* data using random forest, support vector machine, and naïve Bayes classifier. The classifications of the samples are identical for all three methods.

Random forest

		Predicted class		
		bacterial	fungal	mock-infected
**True class**	bacterial	0	0	0
	fungal	0	5	1
	mock-infected	0	0	6

Accuracy: 0.917

Support vector machine

		Predicted class		
		bacterial	fungal	mock-infected
**True class**	bacterial	0	0	0
	fungal	0	5	1
	mock-infected	0	0	6

Accuracy: 0.917

Naïve Bayes classifier

		Predicted class		
		bacterial	fungal	mock-infected
**True class**	bacterial	0	0	0
	fungal	0	5	1
	mock-infected	0	0	6

Accuracy: 0.917

REFERENCES

Liaw, A. and Wiener, M. (2002), Classification and Regression by randomForest, *R News*, 2, 3, 18–22

Meyer, D., Dimitriadou, E., Hornik, K., Weingessel, A., and Leisch, F. (2012), e1071: Misc Functions of the Department of Statistics (e1071), TU Wien
